# Freezing of gait is a risk factor for cognitive decline in Parkinson’s disease

**DOI:** 10.1007/s00415-022-11371-w

**Published:** 2022-09-27

**Authors:** Yi Qu, Jiangting Li, Yupeng Chen, Jingyi Li, Qixiong Qin, Danlei Wang, Jingwei Zhao, Qingmei Yang, Zhijuan Mao, Yongjie Xiong, Zhe Min, Zheng Xue

**Affiliations:** 1 Department of Neurology, Tongji Medical College, Tongji Hospital, Huazhong University of Science and Technology, Wuhan, 430030 China; 2 Department of Pediatrics, Tongji Medical College, Tongji Hospital, Huazhong University of Science and Technology, Wuhan, 430030 China

**Keywords:** Parkinson’s disease, Freezing of gait, Cognitive impairment, Cognitive domains

## Abstract

**Backgrounds:**

Freezing of gait (FOG) and cognitive impairment are serious symptoms of Parkinson’s disease (PD). Understanding the association between FOG and cognition may help formulate specific interventions for PD individuals.

**Objectives:**

We aimed to investigate the associations of cognitive impairment in different domains with FOG status using multiple neuropsychological tests.

**Methods:**

Two cohorts including 691 and 104 participants were recruited from Parkinson’s progression markers initiative (PPMI) and central China, respectively. All participants underwent FOG assessment and neuropsychological tests, and 595 individuals from PPMI and 51 from central China were enrolled for longitudinal observation. Cross-sectional and longitudinal associations between cognition and FOG status were evaluated using multivariable-adjusted models.

**Results:**

Worse cognitive performances were observed in patients with FOG compared to those without FOG in both cohorts (*β* = − 0.020, *p* < 0.001) using multivariate-adjusted models. Moreover, patients with progressive FOG during follow-up manifested more serious cognitive declines (HR = 1.40, 95% CI = 1.07–1.80). The FOG was mainly associated with the decline of executive, attention, and orientation. Furthermore, FOG was associated with higher levels of cognition-related biomarkers including T-tau, P-tau, and NfL in cerebrospinal fluid (*p* < 0.050).

**Conclusions:**

FOG is a risk factor for cognitive decline in PD, which emphasizes the need for early detection and monitoring of cognitive changes and interventions on cognitive impairments in PD patients with FOG.

**Supplementary Information:**

The online version contains supplementary material available at 10.1007/s00415-022-11371-w.

## Introduction

Parkinson’s disease (PD) is the second most common neurodegenerative disease characterized by a broad spectrum of motor and non-motor symptoms with complex clinical, genetic, and molecular features [1]. Cognitive decline is one of the most serious non-motor syndromes of PD, as it is associated with decreased quality of life and increased burden on institutions, economics, and caregivers [2]. It was reported that 25% of PD patients were diagnosed with mild cognitive impairment, and approximately 80% of them would ultimately progress to dementia [1, 3]. Freezing of gait (FOG), a paroxysmal gait disturbance in which patients lose the ability to initiate or resume walking, is a serious symptom of PD and associates with disease severity [4]. The occurrence of FOG and cognitive deficits are highly variable at the different stages of PD, and both jointly lead to poor quality of life [5].

FOG is not a pure motor symptom, but rather a complex interaction effect between motor and cognition [6]. Previous studies have verified that patients with gait disturbances have a greater proportion of cognitive decline and a higher risk of developing mild cognitive impairment or dementia [7, 8], and PD patients with FOG showed faster rates of cognitive decline than those without FOG in a 2-year follow-up study [9]. However, it is reported that cognitive impairment only added the risk of FOG in PD subjects without motor complications [10], and there were studies showing no significant association between FOG and cognition [11, 12]. Thus, the association between FOG and cognitive impairment in PD remains unclear.

We hypothesize that FOG is a risk factor of cognitive impairments in PD patients. To test our hypothesis, we analyzed the association between FOG and cognitive impairment in two cohorts including 691 and 104 participants recruited from Parkinson’s progression markers initiative (PPMI) and central China, respectively. Moreover, 595 individuals from PPMI and 51 from central China were enrolled for longitudinal observation of FOG progression and cognition impairment. Understanding the association between FOG and cognition can help formulate specific interventions for PD individuals with comorbidities of FOG and cognitive impairment.

## Methods

### Study participants

#### PPMI

All clinical information of PPMI in the present study was obtained and used after formal authorization (http://www.ppmi-info.org) [13]. The PPMI was founded by the Michael J. Fox Foundation, which specifically aimed to define reliable biomarkers for predicting PD progression and create a channel for the accelerated blossom and clinical application of novel disease-modifying therapeutics by establishing large-cohort observational studies. Participants were enrolled into PPMI if they (a) were > 30 years, (b) diagnosed with PD in 2 years without medication treating, (c) had at least two symptoms of resting tremor, bradykinesia, and rigidity, or an asymmetric resting tremor or asymmetric bradykinesia, and (d) Hoehn and Yahr stages < 3. Participants were excluded if they (1) were diagnosed with Parkinsonism-plus syndromes, including multiple system atrophy, progressive supranuclear palsy, and Lewy body dementia; (2) had the history of surgery, including stereotactic nerve nuclei lesions and deep brain stimulation; (3) had the history of psychiatric symptoms, cancer, or any serious cardiovascular complications, and (4) could not complete the gait or cognitive evaluation. All patients underwent 3-month intervals follow-up in the first year and 6-month intervals in the subsequent years. The additional follow-up was conducted before the regularly scheduled visit if one withdrew early from the study or began symptomatic therapy. The data up to 10 years of follow-up were included from this study.

#### Central Chinese cohort

The Central Chinese cohort was an observational, single-center, longitudinal study that enrolled PD participants who visited the Department of Neurology, Tongji Hospital, Tongji Medical College, Huazhong University of Science and Technology from October 2014 to October 2021. The diagnosis of PD was based on the clinical diagnostic criteria for movement disorders in 2015 [14], and the and exclusion criteria were same as those used for PPMI. This study was approved by the Medical Ethics Committee of Tongji Hospital (Wuhan, China). Written informed consent was obtained from participants or their legally acceptable representatives. After enrollment, participants were followed up every half to 2 years.

### Clinical assessments

#### FOG assessments

FOG was evaluated using the Movement Disorders Society–Unified Parkinson’s Disease Rating Scale (MDS–UPDRS) item 2.13 (freezing) and item 3.11 (FOG) for PPMI. The present FOG status was defined as any of these two items ≥ 1, while FOG severity was defined as the total score of MDS–UPDRS item 2.13 and 3.11. As well, the progression of FOG was defined as the FOG score ≥ 1 at any point during follow-up periods. On the other hand, the Freezing of Gait Questionnaire (FOG-Q) for the Central Chinese cohort. FOG-Q is a patient-reported outcome measure, which has six questions and can easily be administered in clinics. The total FOG-Q score ≥ 1 was defined as FOG individuals, and higher FOG-Q scores represents the more severity of FOG symptoms [15].

#### Cognitive assessments

Global cognitive functions were assessed by Montreal Cognitive Assessment (MoCA) in PPMI and the Central Chinese cohort. Other cognitive indicators were also examined for several specific areas, including verbal episodic memory (Hopkins Verbal Learning Test [HVLT] Immediate Recall; HVLT Delayed Recall; HVLT Recognition), visuospatial ability (Judgment of Line Orientation [JoLO]), executive function/working memory (Letter Number Sequencing [LNS]), language (Semantic Fluency Test), and processing speed/attention (Symbol Digit Modality Test [SDMT]) [16], as well as subscores of MoCA (visuospatial/executive, naming, attention, language, abstraction, delayed memory and orientation). All the cognitive tests scores were corrected according to the published norms. The cognitive declines were defined as at least two cognitive tests of more than 1.5 standard deviations below normal at baseline in line with described before [17].

#### PD Subtypes and levodopa equivalent daily dose (LEDD) assessments

Tremor dominant (TD) and postural instability and gait difficulty (PIGD) were grouped based on the ratio of TD to PIGD scores: TD patients with ratios > 1.15, PIGD with ratios < 0.90, and unclear with ratios among 0.90–1.15 [18]. LEDD was calculated according to the common conversion formulae [19]. The patients of PPMI did not take anti-PD medicines for their condition at the time of evaluation, while patients in the Central Chinese cohort were on medication.

### Fluid biomarkers measurements

The detailed approaches were as described previously [20]. Cerebrospinal fluid (CSF) amyloid-β_1-42_ (Aβ_1-42_), total-Tau (T-tau), and phosphorylated tau (P-tau) were measured using the xMAP-Luminex platform with INNOBIA AlzBio3 immunoassay kit-based reagents (Fujirebio-Innogenetics, Ghent, Belgium), while Total α-synuclein and NfL levels were measured by enzyme-linked immunosorbent assay kit (Covance, Dedham, MA). Serum neurofilament light (NfL) levels were measured by the Simoa Human NF-light Advantage kit (UmanDiagnostics, Umeå, Sweden) using Single Molecule Array (Simoa) technology. The impacts of possible extreme outliers on the results were weakened through additional quality control.

### Statistical analyses

All analyses were conducted using R (version 3.6.3), and the statistical significance threshold was set at a two-tailed *p* < 0.05.

Differences in demographic characteristics between patients with FOG and without FOG were assessed using the Mann–Whitney *U* test and *χ*^2^ test. Baseline associations of FOG and cognitive impairment were explored by multiple linear regression models that adjusted for age, sex, education levels, disease onset age, disease duration, MDS–UPDRS-III, Hoehn–Yahr stages, Geriatric Depression Scale (GDS) and apathy (MDS–UPDRS-I). The demographic characteristics and variables did not display a normal distribution (Kolmogorov–Smirnov test, *p* < 0.05); therefore, they were log-transformed to approximate the normal distribution by “car” package of R. Cox models were employed to compare the probabilities of cognitive decline with FOG, while mixed-effects linear models were conducted to assess the changes of cognitive impairment and fluid biomarkers. The models included random intercepts and slopes for time and an unstructured covariance matrix for random effects, which was regarded as a predictor of the interactions between time and dependent variables.

## Results

### Participants characteristics of PPMI

Baseline characteristics of the study participants were presented in Table 1. Briefly, 691 participants aged 62.2 years (SD = 10.2; 286 females) from PPMI were enrolled at baseline, therein 76 (11.0%) patients with FOG were identified. Of these, 96 individuals had no available visit data on follow-ups so that were excluded. Finally, 595 patients were included during the follow-ups for the longitudinal analyses, and the average follow-up period was 5.0 ± 2.4 years. Therein, 231 (43.5%) of 531 PD patients without FOG developed to FOG during the 10-year follow-up. There were no significant differences in age, educational levels and disease onset age between the FOG and non-FOG individuals as determined by Mann–Whitney *U* test (*p* > 0.05).

**Table 1:** Characteristics of participants in PPMI

Characteristic	Baseline (*n* = 691)			Longitudinal (*n* = 531)		
	Non-FOG (*n* = 615)	FOG (*n* = 76)	*p* value	Stable (*n* = 300)	Progression (*n* = 231)	*p* value
Demographic characteristics						
Age (SD), year	62.4 (10.0)	61.1 (11.2)	0.247	61.9 (10.3)	62.7 (9.4)	0.576
Female%, *n* (%)	251 (40.1)	35 (46.1)	0.382	139 (46.3)	161 (53.7)	**0.001**
Education (SD), year	15.4 (3.5)	14.9 (4.0)	0.253	15.4 (3.5)	15.4 (3.5)	0.701
Disease onset (SD), year	59.6 (10.4)	56.4 (11.1)	**0.005**	59.0 (10.7)	60.1 (9.8)	0.408
Disease duration (SD), year	2.7 (3.4)	4.7 (3.2)	** < 0.001**	2.9 (4.2)	2.6 (2.4)	0.815
Sub-types (TD/PIGD)	476/79	30/40	** < 0.001**	240/32/28	169/34/28	0.240
Hoehn–Yahr stages	1.6 (0.5)	2.1 (0.6)	** < 0.001**	1.6 (0.5)	1.7 (0.5)	**0.017**
Neuropsychological tests						
MoCA	26.7 (2.7)	25.2 (4.3)	**0.016**	26.9 (2.5)	26.4 (2.9)	**0.004**
HVLT total recall	45.7 (11.1)	43.2 (12.4)	0.266	46.7 (11.2)	44.2 (10.7)	**0.036**
HVLT delayed recall	44.9 (11.6)	42.8 (12.8)	0.310	45.7 (11.7)	43.7 (11.3)	**0.023**
HVLT recognition	45.6 (11.3)	44.0 (12.9)	0.437	46.6 (10.9)	44.2 (11.7)	**0.023**
JoLO	11.6 (3.1)	10.7 (3.4)	**0.050**	12.5 (2.8)	12.3 (3.1)	0.550
LNS	11.2 (3.0)	10.1 (3.4)	**0.024**	11.6 (2.9)	10.8 (3.0)	**0.010**
Semantic fluency test	50.8 (10.2)	47.2 (14.5)	**0.048**	51.0 (9.8)	50.1 (11.0)	0.464
SDMT	44.6 (9.9)	40.1 (44.1)	**0.017**	45.6 (9.4)	43.8 (10.4)	0.081
GDS	5.4 (1.5)	6.1 (1.6)	** < 0.001**	5.4(1.6)	6.1 (1.6)	** < 0.001**
MDS–UPDRS						
UPDRS-I	6.0 (4.4)	10.1 (6.1)	** < 0.001**	5.1 (4.0)	6.5 (4.5)	** < 0.001**
UPDRS-II	5.7 (4.1)	11.9 (5.6)	** < 0.001**	4.5 (3.3)	6.9 (4.3)	** < 0.001**
UPDRS-III	20.4 (9.5)	29.9 (13.1)	** < 0.001**	19.3 (9.1)	21.2 (9.4)	**0.011**
UPDRS-IV	1.0 (1.9)	3.3 (3.4)	** < 0.001**	0.7 (1.5)	1.2 (2.2)	0.231
CSF biomarkers						
Aβ_1-42_, pg/ml (*n* = 473)	893.3 (407.4)	883.1 (454.1)	0.616	922.9 (445.8)	856.1 (365.7)	0.241
T-tau, pg/ml (*n* = 572)	166.9 (61.7)	168.8 (71.1)	0.888	169.0 (63.2)	161.8 (57.6)	0.324
P-tau, pg/ml (*n* = 571)	14.2 (5.6)	14.5 (6.1)	0.991	14.4 (5.8)	13.8 (5.1)	0.269
NfL, pg/ml (*n* = 224)	107.5 (59.1)	79.2 (42.6)	**0.028**	100.8 (52.2)	116.6 (66.7)	0.065
α-Synuclein, pg/ml (*n* = 475)	1507.5 (662.8)	1530.6 (924.6)	0.462	1561.9 (693.1)	1432.6 (621.3)	**0.039**
Serum NfL, pg/ml (*n* = 562)	13.7 (7.5)	15.3 (11.7)	0.826	12.8 (6.3)	14.3 (8.0)	**0.047**

The bold values were symbolized to statistically significant difference (*p* < 0.05)

*Aβ*_*1-42*_ amyloid β_1-42_, *CSF* cerebrospinal fluid, *FOG-Q* Freezing of Gait Questionnaire, *GDS* Geriatric Depression Scale, *HVLT* Hopkins verbal learning test, *JoLO* Benton judgment of line orientation, *LNS* letter number sequencing, *MDS–UPDRS* Movement Disorders Society Unified Parkinson’s Disease Rating Scale, *MoCA* Montreal cognitive assessment, *n* number, *NfL* neurofilament light, *SD* standard deviation, *SDMT* symbol digit modality test, *PIGD* postural instability and gait difficulty, *PPMI* Parkinson’s progression markers initiative, *P-tau* phosphorylated tau, *TD* tremor dominant, *T-tau* total tau

### Baseline associations of FOG with cognitive functions of PPMI

Using the multiple linear regression models, we found that participants with FOG status had lower scores of MoCA (*β* = − 0.020, *p* < 0.001), LNS (*β* = − 0.050, *p* = 0.032) and Semantic Fluency Test (*β* = − 0.030, *p* = 0.036) after adjusting for age, sex, educational levels, age at disease onset, disease − duration, MDS-UPDRS-III, Hoehn-Yahr stages, GDS and apathy. This suggests that individuals with FOG had worse cognitive performances compared to those without FOG. Specifically, FOG was mainly associated with the cognitive functions of visuospatial/executive (*β* = − 0.095, *p* < 0.001), attention (*β* = − 0.037, *p* = 0.021), abstraction (*β* = − 0.070, *p* < 0.001) and orientation (*β* = − 0.013, *p* = 0.017) using MoCA subscores. Similar to the results of FOG status, higher FOG scores were also significantly associated with JoLO (*β* = − 0.019, *p* = 0.038) and SDMT (*β* = − 0.021 *p* = 0.002). There were no significant associations between FOG and other neuropsychological tests and fluid biomarkers (Table 2).

**Table 2:** Associations of FOG with MMSE and MoCA using multiple linear regression in PPMI

Cognitive measures	FOG status						FOG severity					
	*β*	SE	*F*	*df*	*p* value	*R*^2^	*β*	SE	*F*	*df*	*p* value	*R*^2^
MoCA (*n* = 691)												
Total score	− **0.020**	**0.007**	**14.6**	**681**	** < 0.001**	**0.151**	− **0.010**	**0.003**	**14.6**	**681**	** < 0.001**	**0.151**
Visuospatial/executive	− **0.144**	**0.039**	**14.4**	**681**	** < 0.001**	**0.149**	− **0.095**	**0.019**	**16.0**	**681**	** < 0.001**	**0.163**
Naming	− 0.006	0.020	4.2	681	0.759	0.046	− 0.001	0.010	4.2	681	0.887	0.041
Attention	− **0.074**	**0.033**	**6.6**	**681**	**0.026**	**0.069**	− **0.037**	**0.016**	**8.4**	**681**	**0.021**	**0.069**
Language	− 0.029	0.054	8.8	681	0.584	0.088	− 0.020	0.026	9.0	681	0.448	0.088
Abstraction	− **0.144**	**0.041**	**5.7**	**681**	** < 0.001**	**0.058**	− **0.070**	**0.020**	**9.1**	**681**	** < 0.001**	**0.056**
Delayed memory	− 0.068	0.087	9.2	681	0.438	0.097	0.020	0.043	10.4	681	0.638	0.096
Orientation	− 0.019	0.011	4.5	681	0.100	0.043	**0.013**	**0.006**	**4.9**	**681**	**0.017**	**0.048**
HVLT (*n* = 634)												
HVLT total recall	-0.018	0.015	13.7	624	0.243	0.153	− 0.010	0.007	13.8	624	0.182	0.154
HVLT delay recall	− 0.016	0.017	10.8	624	0.336	0.122	− 0.003	0.080	10.7	624	0.677	0.121
HVLT recognition	− 0.015	0.017	6.4	624	0.382	0.072	− 0.001	0.008	6.3	624	0.886	0.071
JoLO (*n* = 631)	− 0.024	0.019	9.5	621	0.507	0.109	− **0.019**	**0.009**	**10.0**	**621**	**0.038**	**0.114**
LNS (*n* = 631)	− **0.050**	**0.019**	**13.7**	**621**	**0.032**	**0.153**	− **0.031**	**0.009**	**14.7**	**621**	** < 0.001**	**0.164**
Semantic fluency test (*n* = 633)	− **0.030**	**0.014**	**5.0**	**623**	**0.036**	**0.054**	− **0.016**	**0.007**	**4.8**	**623**	**0.093**	**0.051**
SDMT (*n* = 633)	− 0.024	0.014	10.2	622	0.095	0.116	− **0.021**	**0.007**	**11.1**	**622**	**0.002**	**0.126**
CSF biomarkers												
Aβ1-42 (*n* = 473)	− 0.032	0.077	0.9	456	0.677	− 0.001	− 0.009	0.052	0.9	456	0.858	-0.001
T-tau (*n* = 572)	**− 0.001**	**0.009**	**6.7**	**556**	**0.923**	**0.083**	**0.002**	**0.005**	**6.7**	**556**	**0.617**	**0.083**
P-tau (*n* = 571)	0.001	0.013	6.6	556	0.925	0.082	0.005	0.007	6.7	556	0.475	0.083
NfL (*n* = 224)	**− 0.097**	**0.111**	**16.8**	**209**	**0.236**	**0.394**	**− 0.109**	**0.098**	**16.8**	**209**	**0.269**	**0.395**
α-Synuclein (*n* = 475)	− 0.049	0.074	2.6	458	0.505	0.031	− 0.018	0.050	2.6	458	0.716	0.030
Serum NfL (*n* = 562)	**0.046**	**0.052**	**53.3**	**542**	**0.375**	**0.461**	**0.039**	**0.029**	**53.5**	**542**	**0.180**	**0.462**

All models were adjusted for age, sex, education level, age at disease onset, disease duration, MDS–UPDRS-III, Hoehn–Yahr stages, GDS and apathy (MDS–UPDRS-I)

The bold values were symbolized to statistically significant difference (*p* < 0.05)

All *R*^2^ was adjusted *R*^2^

*Aβ*_*1-42*_ amyloid β_1-42_, *CSF* cerebrospinal fluid, *df* degree of freedom, *FOG* Freezing of gait, *GDS* Geriatric Depression Scale, *HVLT* Hopkins Verbal Learning Test, *JoLO* Benton Judgment of Line Orientation, *LNS* Letter Number Sequencing, Movement Disorders Society Unified Parkinson’s Disease Rating Scale, *MoCA* Montreal Cognitive Assessment, *n* number, *NfL* neurofilament light, *SDMT* Symbol Digit Modality Test, *PPMI* Parkinson’s progression markers initiative, *P-tau* phosphorylated tau, *SE* standard error, *T-tau* total tau

### Longitudinal associations of FOG with cognitive functions of PPMI

#### Associations of baseline FOG and cognitive changes

**Fig. 1:**
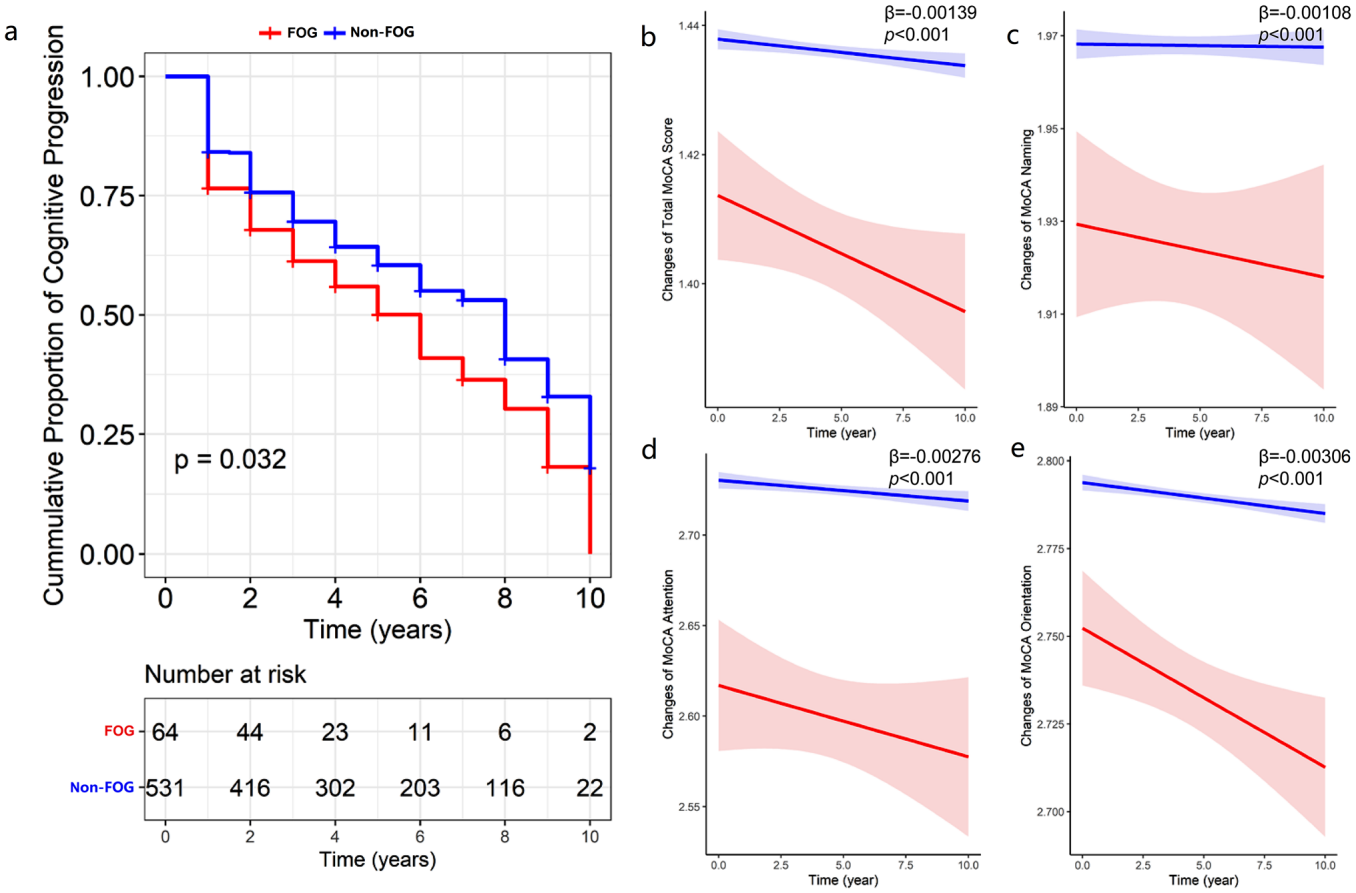
Associations between Baseline FOG and longitudinal cognitive changes in PPMI. The Kaplan–Meier survival curve did not show that patients with FOG had a higher risk of cognitive impairment progression (log-rank *p* = 0.032, **a**). Mixed-effect linear models indicated that baseline individuals with FOG had greater cognitive decline, including total MoCA (*β* = − 0.00139, *p* < 0.001, **b**), naming (*β* = − 0.00108, *p* < 0.001, **c**), attention (*β* = − 0.00276, *p* < 0.001, **d**) and orientation (*β* = − 0.00306, *p* < 0.001, **e**). FOG, freezing of gait; MoCA, Montreal cognitive assessment; PPMI, Parkinson’s progression markers initiative

**Fig. 2:**
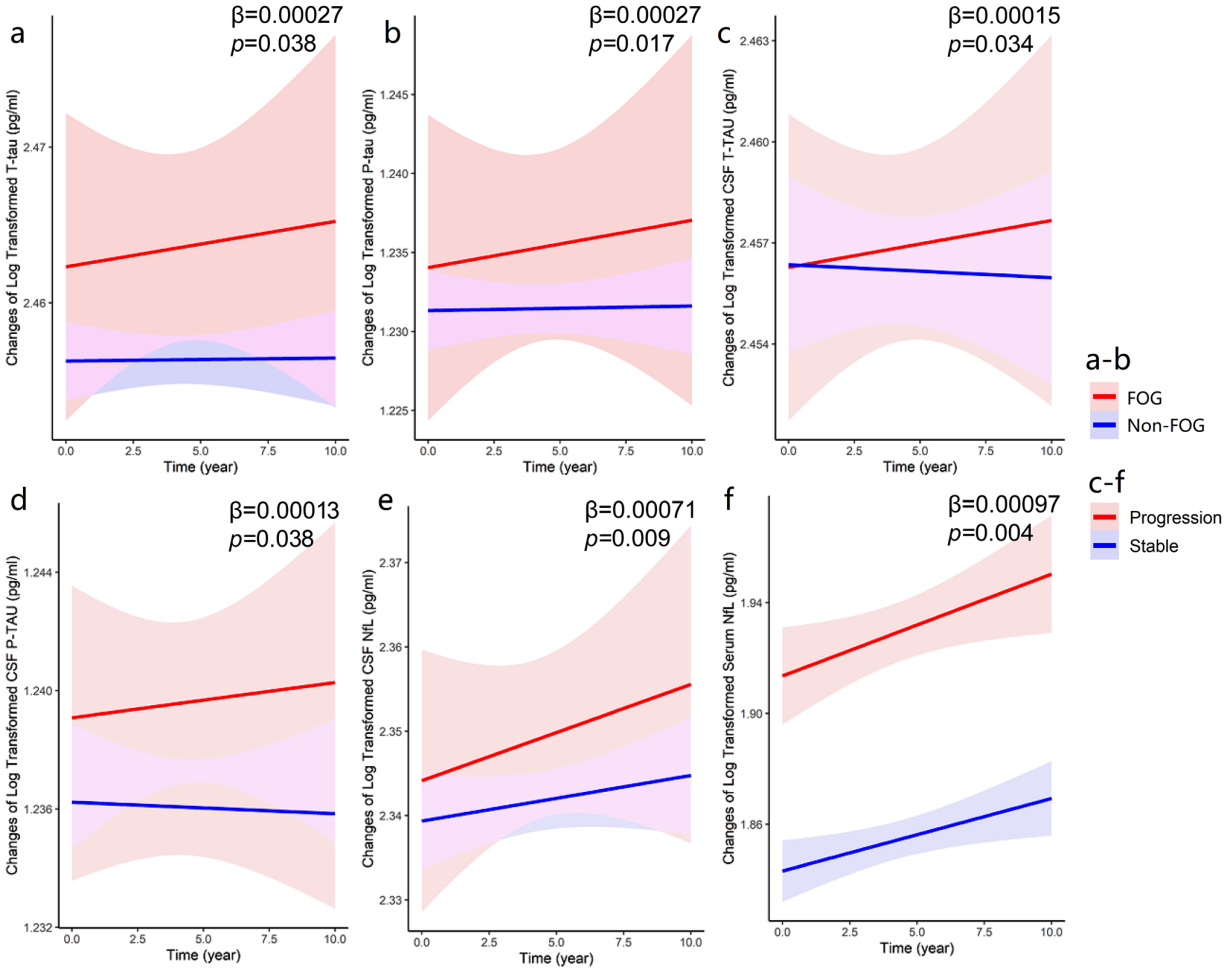
Associations between FOG and longitudinal changes of fluid biomarkers in PPMI. On one hand, baseline FOG patients had greater accumulation of CSF T-tau (*β* = 0.00027, *p* = 0.038, **a**) and P-tau (*β* = 0.00027, *p* = 0.017, **b**), which might represent the worse cognitive condition. On the other hand, PD patients with FOG progression were likely associated more increases of CSF T-tau (*β* = 0.00015, *p* = 0.034, **c**), P-tau (*β* = 0.00013, *p* = 0.038, **d**), NfL (*β* = 0.00071, *p* = 0.009, **e**), as well as serum NfL (*β* = 0.00097, *p* = 0.004, **f**). CSF, cerebrospinal fluid; FOG, freezing of gait; NfL, neurofilament light; T-tau, total tau; PPMI, Parkinson’s progression markers initiative; P-tau, phosphorylated tau

We analyzed the association between baseline FOG and cognitive decline using multivariable-adjusted Cox proportional hazard regression and mixed-effect linear models. Patients with FOG were at a higher risk of cognitive decline (hazard ratio [HR] = 1.53, 95% confidence interval [CI] = 1.03–2.27, *p* = 0.035, Fig. 1a) compared to non-FOG subjects. Patients with FOG also had greater declines of total MoCA score (*β* = − 0.00138, *p* < 0.001, Fig. 1b), as well as naming (*β* = − 0.00108, *p* < 0.001, Fig. 1c), attention (*β* = − 0.00276, *p* < 0.001, Fig. 1d) and orientation (*β* = − 0.00306, *p* < 0.001, Fig. 1e) of MoCA than non-FOG subjects. In addition, the occurrence of FOG at baseline were associated with greater accumulation cognitive impairment-related biomarkers including T-tau (*β* = 0.00027, *p* = 0.038, Fig. 2a) and P-tau (*β* = 0.00027, *p* = 0.017, Fig. 2b) in CSF. On the other hand, the baseline FOG severity was associated with decreased visuospatial/executive (*β* = − 0.00131, *p* = 0.036), language (β = -0.00158, *p* = 0.007) and delayed memory (*β* = − 0.00096, *p* = 0.002) in MoCA, as well as LNS (*β* = − 0.00170, *p* = 0.049) (Table 3).

**Table 3:** Associations between FOG progression and cognitive changes by mixed-effects linear model? in PPMI

Cognitive measures	Baseline FOG status				Baseline FOG severity				Longitudinal FOG progression (*n* = 531)			
	*β*	SE	*df*	*p* value	*β*	SE	*df*	*p* value	*β*	SE	*df*	*p* value
MoCA (*n* = 595)												
Total score	− **0.00139**	**0.00032**	**382.4**	** < 0.001**	− **0.00094**	**0.00016**	**512.3**	** < 0.001**	− **0.00040**	**0.00014**	**192.9**	**0.005**
Visuospatial/executive	− 0.00153	0.00104	398.8	0.143	− **0.00131**	**0.00062**	**705.0**	**0.036**	− **0.00244**	**0.00055**	**276.7**	** < 0.001**
Naming	− **0.00108**	**0.00030**	**165.9**	** < 0.001**	− **0.00112**	**0.00021**	**434.0**	** < 0.001**	− 0.00027	0.00014	196.5	0.053
Attention	− **0.00276**	**0.00073**	**335.2**	** < 0.001**	− **0.00199**	**0.00043**	**592.8**	** < 0.001**	− **0.00085**	**0.00035**	**246.4**	**0.014**
Language	− 0.00106	0.00087	325.4	0.225	− **0.00158**	**0.00058**	**750.5**	**0.007**	− 0.00062	0.00045	255.1	0.174
Abstraction	− 0.00035	0.00070	400.0	0.618	− 0.00364	0.00044	850.3	0.413	− 0.00051	0.00038	284.0	0.176
Delayed memory	− 0.00244	0.00142	323.9	0.086	− **0.00300**	**0.00096**	**758.8**	**0.002**	− **0.00166**	**0.00073**	**253.2**	**0.023**
Orientation	− **0.00306**	**0.00071**	**272.1**	** < 0.001**	− **0.00232**	**0.00038**	**408.7**	** < 0.001**	− **0.00074**	**0.00024**	**117.9**	**0.002**
HVLT (*n* = 586)												
HVLT total recall	− 0.00024	0.00026	426.7	0.352	− 0.00021	0.00018	930.2	0.246	− **0.00033**	**0.00013**	**308.5**	**0.017**
HVLT delay recall	− 0.00022	0.00030	431.8	0.477	− 0.00032	0.00021	934.9	0.124	− **0.00052**	**0.00015**	**306.8**	** < 0.001**
HVLT recognition	− 0.00044	0.00032	452.9	0.191	− 0.00005	0.00023	930.1	0.829	− 0.00025	0.00017	324.8	0.144
JoLO (*n* = 582)	− 0.00044	0.00106	424.2	0.681	− 0.00325	0.00075	909.1	0.664	− **0.00042**	**0.00011**	**300.5**	** < 0.001**
LNS (*n* = 583)	− 0.00028	0.00137	389.5	0.839	− **0.00170**	**0.00086**	**767.3**	**0.049**	− **0.00066**	**0.00022**	**268.9**	**0.003**
Semantic fluency test (*n* = 587)	− 0.00009	0.00025	408.1	0.727	− 0.00018	0.00017	896.5	0.288	− **0.00063**	**0.00013**	**267.3**	** < 0.001**
SDMT (*n* = 584)	− 0.00043	0.00028	483.4	0.126	− 0.00031	0.00017	906.2	0.077	− **0.00060**	**0.00015**	**325.0**	** < 0.001**
CSF biomarkers												
Aβ_1-42_ (*n* = 379)	0.00093	0.00178	253.6	0.601	0.00099	0.00162	257.2	0.542	− 0.00016	0.00054	211.5	0.772
T-tau (*n* = 469)	**0.00027**	**0.00013**	**274.5**	**0.038**	**0.00022**	**0.00011**	**382.3**	**0.047**	**0.00015**	**0.00007**	**271.0**	**0.034**
P-tau (*n* = 468)	**0.00027**	**0.00011**	**316.6**	**0.017**	**0.00018**	**0.00009**	**442.8**	**0.048**	**0.00013**	**0.00064**	**272.5**	**0.038**
NfL (*n* = 224)	0.00007	0.00042	164.5	0.870	0.00027	0.00037	162.6	0.457	**0.00071**	**0.00027**	**154.1**	**0.009**
α-Synuclein (*n* = 380)	− 0.00040	0.00179	1044	0.823	− 0.00058	0.00163	1046	0.721	0.00059	0.00120	968.1	0.623
Serum NfL (*n* = 561)	0.00111	0.00057	506.3	0.051	0.00054	0.00039	901.8	0.173	**0.00097**	**0.00034**	**352.8**	**0.004**

The bold values were symbolized to statistically significant difference (*p* < 0.05)

*All models were adjusted for age, sex, education levels, disease onset age, disease duration, Hoehn–Yahr stages, GDS and apathy (MDS–UPDRS-I)

*Aβ*_*1-42*_ amyloid β_1-42_, *CSF* cerebrospinal fluid, *df* degree of freedom, *FOG* freezing of gait, GDS Geriatric Depression Scale, *HVLT* Hopkins verbal learning test, *JoLO* Benton judgment of line orientation, *LNS* letter number sequencing, Movement Disorders Society Unified Parkinson’s Disease Rating Scale, *MoCA* Montreal cognitive assessment, *n* number, *NfL* neurofilament light, *SDMT* symbol digit modality test, *PPMI* Parkinson’s progression markers initiative, *P-tau* phosphorylated tau, SE standard error, *T-tau* total tau

#### Associations of FOG progression and cognitive changes

**Fig. 3:**
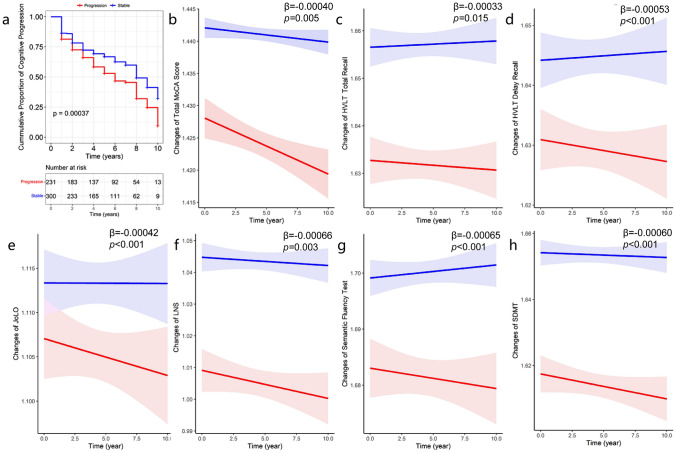
Associations between FOG progression with longitudinal cognitive changes in PPMI. The Kaplan–Meier survival curve expressed that patients with FOG progression had a higher risk of cognitive decline (log-rank *p* = 0.00037, **a**). Mixed-effect linear models indicated that the progression of FOG meant larger decreases of cognition, including total MoCA (*β* = − 0.00040, *p* = 0.005, **b**), HVLT total recall (*β* = − 0.00033, *p* = 0.015, **c**), HVLT delayed recall (*β* = − 0.00053, *p* < 0.001, **d**), JoLO (*β* = − 0.00042, *p* < 0.001, **e**), LNS (*β* = − 0.00066, *p* = 0.003, **f**), Semantic Fluency Test (*β* = − 0.00065, *p* < 0.001, **g**) and SDMT (*β* = − 0.00060, *p* < 0.001, **h**). FOG, freezing of gait; HVLT, Hopkins verbal learning test; JoLO, Benton judgment of line orientation; LNS, letter number sequencing; MoCA, Montreal cognitive assessment; PPMI, Parkinson’s progression markers initiative; SDMT, symbol digit modality test

Cox models showed that patients with FOG progression had higher risk of cognitive impairment (HR = 1.40, 95% CI = 1.07–1.80, *p* = 0.007, Fig. 3a). Patients with FOG progression had more decreases of MoCA scores (*β* = − 0.00040, *p* = 0.005; Fig. 3b), HVLT total recall (*β* = − 0.00033, *p* = 0.015; Fig. 3c), HVLT delayed recall (*β* = − 0.00052, *p* < 0.001; Fig. 3d), JoLO (*β* = − 0.00042, *p* < 0.001; Fig. 3e), LNS (*β* = − 0.00066, *p* = 0.003; Fig. 3g), Semantic Fluency Test (*β* = − 0.00063, *p* < 0.001; Fig. 3f) and SDMT (*β* = − 0.00060, *p* < 0.001; Fig. 3h). The separate domains analyses revealed that the FOG progression was mostly correlated with visuospatial/executive (*β* = − 0.00244, *p* < 0.001), attention (*β* = − 0.00085, *p* = 0.014), delayed memory (*β* = − 0.00166, *p* = 0.023) and orientation (*β* = − 0.00074, *p* = 0.002) of MoCA (Table 3). Furthermore, FOG progression was associated with higher levels of cognitive impairment-related biomarkers including CSF T-tau (*β* = 0.00015, *p* = 0.034, Fig. 2c), P-tau (*β* = 0.00013, *p* = 0.038, Fig. 2d), NfL (*β* = 0.00071, *p* = 0.009, Fig. 2e) and serum NfL (*β* = 0.00097, *p* = 0.004, Fig. 2f). These data suggest that FOG progression is associated with cognitive impairment.

### Associations of FOG with cognitive functions in the central Chinese cohort

We next assessed the association between FOG and cognitive functions in another cohort including 104 PD patients from central China, of which 63 (60.6%) patients with FOG were identified (eTable 1). Therein, 51 (49.0%) participants were included into longitudinal analyses with an average follow-up of 2.7 ± 1.0 years. Twenty-five non-FOG subjects were conducted to explore the association between FOG progression and cognitive decline, and 6 (24.0%) subjects progressed to FOG. There were no significant differences in demographic characteristics between FOG and non-FOG patients (all *p* > 0.05).

In the Central Chinese cohort, FOG was evaluated using FOG-Q. The presence of baseline FOG was associated with declines of MoCA (*β* = − 0.085, *p* < 0.001) and China-Modified Mini-Mental State Examination (CM-MMSE; *β* = − 0.036, *p* = 0.037) according to multivariable-adjusted multiple linear regression models. The worse cognitive functions were more frequently observed in terms of naming, attention, calculation, and delayed memory in subdomains, which showed similar results with continuous FOG-Q scores (eTable 2). On the other hand, the higher FOG-Q scores were associated with greater decreases of MoCA (*β* = − 0.00056, *p* = 0.001) and CM-MMSE (*β* = − 0.00027, *p* = 0.034), while FOG progression was related to MoCA (*β* = − 0.00323, *p* < 0.001) and CM-MMSE (*β* = − 0.00213, *p* < 0.001) (eTable 3). The mainly involved domains of cognition were delayed memory and orientation.

## Discussion

The present study comprehensively evaluated the associations between FOG and cognitive functions in two prospective cohorts of PD patients. In support of the hypothesis that FOG is a risk factor of cognitive impairment, worse cognitive performances were observed in patients with FOG compared to those without FOG in both cohorts. Moreover, patients with progressive FOG during follow-ups manifested more serious cognitive declines. Of note, FOG was associated with higher levels of cognitive impairment-related biomarkers in CSF and serum. These findings deepen the understanding of the associations between FOG and cognitive impairment, and emphasize that more attention should be paid to cognitive changes in PD patients with FOG.

The primary results demonstrated that patients with FOG have worse cognitive performances at baseline and greater cognitive decline at follow-ups compared to patients without FOG. The presence of FOG and FOG progression were associated with deficits in global cognition and specific domains. Our findings are consistent with previous studies that patients with FOG have higher risk and greater decline of cognitive impairment than those without FOG [7–9]. However, there are reports showing no significant associations between FOG and cognitive impairment [11, 12]. The possible explanations include the interference of motor function and insensitive cognitive scales [11]. Several studies have reported that dopaminergic nuclei is associated with cognitive impairment as the major impaired lesions of FOG [21]. The diffuse destruction of nigrostriatal and extra-nigrostriatal pathways also play vital roles in the relation between FOG and cognition performances [22]. Several functional magnetic resonance imaging (fMRI) studies discover the reduced structural connectivity in FOG patients between pedunculopontine nucleus and cerebellum [23], thalamus and frontal regions, as well as prefrontal cortex [24]. Furthermore, one multi-tracer positron emission tomography study demonstrates that cortical cholinergic denervation is linked with elevated risk of FOG, especially in patients with concomitant cortical amyloid deposition [25]. These studies suggest the significant roles of FOG in the pathogenesis of cognitive impairment. Nevertheless, it is also raised that FOG occurs via two parallel processes of increasing motor severity and advancing cognitive impairment [12]. Besides, the more serious motor symptoms in FOG group might result in poor performances of speech or writing when making neurophysiological tests. In consequence, it could not reflect the cognitive performances truthfully. In our study, significant correlations survived after adjusting multivariable analysis suggesting that FOG is a risk factor for cognitive impairment.

Fluid biomarkers are valuable and sensitive in early detection of central pathology. Interestingly, our data show that the progression of FOG was significantly associated with cognition-related biomarkers including T-tau, P-tau and NfL in CSF, as well as NfL in serum. This is consistent with previous reports that elevated levels of CSF P-tau and serum NfL accompanied the pathogenesis of cognitive impairment in FOG patients [26, 27]. *Apolipoprotein E* (*APOE*) *ε4* genotype was reported to be associated with faster FOG progression in PD patients, suggesting a novel genetic risk factor for FOG [28]. The *APOE ε4* carriers have higher levels of tau pathology than non-carriers, suggesting that this genotype may affect the neural circuitry associated with FOG and needs further study.

FOG is provoked by the deficits of executive and attention when passing narrow spaces or turning [29]. Structural and functional changes of frontal regions affect both executive and attention [30, 31]. Our findings indicated that FOG was a risk factor for cognitive impairment in individuals with PD, especially in specific cognitive domains of executive and attention. In support of this, neuroimaging studies revealed impairment of executive-attention and visual neural networks in patients with FOG [32], as well as the lower gray volumes or atrophy of frontal and parietal related to executive and visuospatial functions in FOG subjects [33, 34]. Controversies persist about the relationship between FOG and dopaminergic medication. FOG is generally responsive to dopaminergic medication in the most common dopamine-responsive patients, while long-term levodopa treatment may cause FOG deterioration [35, 36]. Levodopa-unresponsive FOG is reported to be associated with executive and visuospatial dysfunction [37]. Levodopa-unresponsive FOG is related to frontostriatal pathway, while levodopa-responsive FOG is associated with posterior cortical regions involved hallucinations. These studies warrant the explorations of FOG drug responsiveness, and more prospective studies are needed to clarify these complex relationships.

Previous studies focused on the cognitive status [10, 38], but PD patients at early stage have slight cognitive decline which may not be detected using simple cognitive classification. The MoCA used in the present study is a sensitive scale for the early detection and diagnosis of cognition impairment [39]. In our central China cohort, FOG-Q was used to evaluate FOG severity and FOG-Q assessment could provide more precise information of the patients with FOG compared to PPMI cohort [15]. More serious FOG is associated with poorer cognitive performances in our cohort, which is consistent with previous studies [40]. A longer duration of follow-up visit of our cohort is in progress to further observe the association between FOG and cognition impairment.

Our study has several limitations. First, the diagnosis of FOG based on the MDS–UPDRS item 2.13 and 3.11 in PPMI was subjective and can be affected by the researchers’ experience. New assessment tools including wearable and virtual reality equipment may be promising. Second, it remained a challenge to eliminate the interfering factors, such as disease duration and severity, and to control motor function discrepancy between FOG and non-FOG groups.

Taken together, our findings suggest that FOG is a risk factor for cognitive impairment in patients with PD. This emphasizes the need for early detection and monitoring of cognitive changes and interventions on cognitive impairments in PD patients with FOG.

## Supplementary Information

Below is the link to the electronic supplementary material.

Supplementary file1 (DOCX 45 KB)

## Data Availability

The clinical data used in this study from PPMI cohort are available at the PPMI website (https://www.ppmi-info.org/access-data-specimens/download-data/), and the data from Central Chinese cohort can be shared on reasonable requests by contacting the corresponding authors.
