# Supplementary material for: Freezing of gait is a risk factor for cognitive decline in Parkinson’s disease
Source: J Neurol. 2022 Sep 27;270(1):466–76. doi: 10.1007/s00415-022-11371-w (PMC9813160; doi:10.1007/s00415-022-11371-w)
Supplement: Supplementary file 1 — Supplementary file1 (DOCX 45 KB) [file 415_2022_11371_MOESM1_ESM.docx]

**SUPPLEMENTARY MATERIAL**

**Title: Freezing of Gait Can Predict Cognitive Decline in Parkinson’s Disease**

Yi Qu^1^, Jiangting Li^1^, Yupeng Chen^1^, Jing-Yi Li^1^, Qixiong Qin^1^, Danlei Wang^1^, Jingwei Zhao^1^, Qingmei Yang^2^, Zhijuan Mao^1^, Yongjie Xiong^1^, Zhe Min^1^, Zheng Xue^1,*^

^1^ Department of Neurology, Tongji Hospital, Tongji Medical College, Huazhong University of Science and Technology, Wuhan, China.

^2^ Department of Pediatrics, Tongji Hospital, Tongji Medical College, Huazhong University of Science and Technology, Wuhan, China.

Corresponding Author:

^*^Address correspondence to Prof. Zheng Xue, Department of Neurology, Tongji Hospital, Tongji College of Medicine, Huazhong University of Science and Technology, Wuhan, 430030, China.

E-mail address: xuezheng@hust.edu.cn

**CONTENT**

| **TITLE** | **PAGE** |
| --- | --- |
| eTable 1. Characteristics of participants in the Chinese cohort. | 1 |
| eTable 2. Associations of FOG with cognition using multiple linear regression in the Chinese cohort. | 2 |
| eTable 3. Associations between FOG progression and cognitive changes by mix-linear models in the Chinese cohort. | 3 |

**eTable 1. Characteristics of participants in the Chinese cohort.**

| Characteristic | Baseline (n=104) | | | Longitudinal (n=25) | | |
| --- | --- | --- | --- | --- | --- | --- |
|  | Non-FOG (n=41) | FOG  (n=63) | *p*-value | Stable (n=19) | Progression (n=6) | *p*-value |
| Demographic characteristics |  |  |  |  |  |  |
| Age (SD), year | 57.7 (10.6) | 62.3 (9.4) | **0.023** | 60.8 (8.7) | 61.3 (13.5) | 0.9266 |
| Female%, n (%) | 16 (39.0) | 31 (49.2) | 0.310 | 9 (47.4) | 4 (75.0) | 0.409 |
| Education (SD), year | 10.2 (4.0) | 7.9 (4.5) | 0.020 | 10.4 (4.8) | 8.7 (5.4) | 0.598 |
| Disease onset (SD), year | 52.5 (10.9) | 55.6 (10.6) | 0.149 | 55.9 (8.0) | 55.2 (13.6) | 0.733 |
| Disease duration (SD), year | 5.2 (4.3) | 6.2 (4.6) | 0.128 | 4.9 (4.7) | 6.2 (5.0) | 0.687 |
| Subtypes (TD/PIGD/unclear) | 16/24/1 | 11/50/2 | **0.050** | 8/11/0 | 4/2/0 | 0.294 |
| Hoehn & Yahr stages | 1.9 (0.7) | 2.7 (0.9) | **<0.001** | 1.9 (0.7) | 2.0 (0.6) | 0.780 |
| LEDD | 506.6 (206.1) | 642.6 (263.4) | **0.010** | 504.8 (191.0) | 695.5 (297.5) | 0.333 |
| Neuropsychological tests |  |  |  |  |  |  |
| CM-MMSE | 27.0 (2.5) | 23.4 (4.8) | **<0.001** | 27.3 (2.2) | 28.5 (1.4) | 0.303 |
| MoCA | 23.8 (3.7) | 18.7 (5.0) | **<0.001** | 23.9 (3.5) | 22.5 (3.2) | 0.251 |
| HAMD | 9.6 (8.4) | 15.4 (7.8) | **<0.001** | 10.1 (8.0) | 17.5 (7.0) | **0.001** |
| MDS-UPDRS |  |  |  |  |  |  |
| UPDRS-Ⅰ | 7.2 (5.5) | 12.3 (6.4) | **<0.001** | 6.1 (4.7) | 10.5 (7.1) | 0.138 |
| UPDRS-Ⅱ | 8.0 (5.9) | 15.7 (7.6) | **<0.001** | 7.2 (7.0) | 9.7 (6.8) | 0.333 |
| UPDRS-Ⅲ | 27.4 (14.4) | 16.2 (7.7) | **<0.001** | 32.5 (18.5) | 32.3 (14.7) | 0.975 |
| UPDRS-Ⅳ | 0.1 (0.7) | 2.2 (4.0) | **0.003** | 0 (0) | 0 (0) | - |
| FOG-Q | 0 (0) | 10.8 (5.0) | **<0.001** | 0 (0) | 0 (0) | **-** |

*Abbreviations:* CM-MMSE, China-Modified Mini-Mental State Examination; FOG-Q, Freezing of Gait Questionnaire; HAMD, Hamilton Depression Scale; MDS-UPDRS, Movement Disorders Society Unified Parkinson’s Disease Rating Scale; MoCA, Montreal Cognitive Assessment; LEDD, levodopa equivalent daily dose; PIGD, postural instability and gait difficulty; SD, Standard Deviation; TD, tremor dominant.

**eTable 2. Associations of FOG with cognition using multiple linear regression in the Chinese cohort.**

| Cognitive measures | FOG status | | | | | | FOG severity | | | | | |
| --- | --- | --- | --- | --- | --- | --- | --- | --- | --- | --- | --- | --- |
|  | β | SE | F | df | *p*-value | R^2^ | β | SE | F | df | *p*-value | R^2^ |
| MoCA |  |  |  |  |  |  |  |  |  |  |  |  |
| Total score | **-0.085** | **0.025** | **7.6** | **83** | **0.001** | **0.414** | **-0.021** | **0.008** | **6.9** | **83** | **0.009** | **0.388** |
| Visuospatial/executive | -0.040 | 0.053 | 3.3 | 83 | 0.450 | 0.194 | -0.013 | 0.016 | 3.3 | 83 | 0.398 | 0.200 |
| Naming | **-0.076** | **0.033** | **2.8** | **83** | **0.025** | **0.161** | **-0.020** | **0.010** | **2.6** | **83** | **0.049** | **0.149** |
| Attention | **-0.090** | **0.032** | **4.4** | **83** | **0.005** | **0.268** | **-0.023** | **0.010** | **4.0** | **83** | **0.018** | **0.247** |
| Language | -0.056 | 0.037 | 3.4 | 83 | 0.132 | 0.206 | -0.013 | 0.011 | 3.3 | 83 | 0.237 | 0.198 |
| Abstraction | -0.078 | 0.043 | 3.1 | 83 | 0.077 | 0.181 | -0.014 | 0.013 | 2.8 | 83 | 0.300 | 0.160 |
| Delayed memory | **-0.166** | **0.058** | **4.2** | **83** | **0.005** | **0.259** | **-0.045** | **0.017** | **4.0** | **83** | **0.012** | **0.245** |
| Orientation | -0.021 | 0.018 | 2.8 | 83 | 0.235 | 0.161 | -0.003 | 0.005 | 2.7 | 83 | 0.524 | 0.151 |
| CM-MMSE |  |  |  |  |  |  |  |  |  |  |  |  |
| Total score | **-0.036** | **0.017** | **7.1** | **88** | **0.037** | **0.385** | **-0.011** | **0.005** | **7.2** | **88** | **0.032** | **0.389** |
| Executive | -0.006 | 0.019 | 4.3 | 88 | 0.744 | 0.251 | -0.002 | 0.006 | 4.3 | 88 | 0.664 | 0.252 |
| Memory | -0.020 | 0.012 | 1.1 | 88 | 0.098 | 0.005 | -0.006 | 0.003 | 1.1 | 88 | 0.096 | 0.005 |
| Attention and Calculation | **-0.167** | **0.046** | **5.6** | **88** | **<0.001** | **0.317** | **-0.045** | **0.014** | **5.2** | **88** | **0.001** | **0.302** |
| Recall | -0.053 | 0.044 | 1.0 | 88 | 0.232 | -0.005 | -0.004 | 0.013 | 0.8 | 88 | 0.788 | -0.002 |
| Language and Praxis | -0.019 | 0.024 | 4.6 | 88 | 0.420 | 0.270 | -0.011 | 0.007 | 4.9 | 88 | 0.124 | 0.284 |

All models were adjusted for age, sex, education level, age at disease onset, disease duration, MDS-UPDRS-Ⅲ, Hoehn & Yahr stages, LEDD, HAMD and apathy (MDS-UPDRS-Ⅰ).

All R^2^ was adjusted R^2^.

*Abbreviations:* CM-MMSE, China-Modified Mini-Mental State Examination; df, degree of freedom; FOG, Freezing of gait; HAMD, Hamilton Depression Scale; LEDD, levodopa equivalent daily dose; MDS-UPDRS, Movement Disorders Society Unified Parkinson’s Disease Rating Scale; MoCA, Montreal Cognitive Assessment; SE, standard error.

**eTable 3. Associations between FOG progression and cognitive changes by mix-linear models in the Chinese cohort.**

| Cognitive measures | Baseline FOG status | | | | Baseline FOG severity | | | | Longitudinal FOG progression | | | |
| --- | --- | --- | --- | --- | --- | --- | --- | --- | --- | --- | --- | --- |
|  | β | SE | df | p-value | β | SE | df | p-value | β | SE | df | p-value |
| MoCA (n=51) |  |  |  |  |  |  |  |  |  |  |  |  |
| Total score | -0.00104 | 0.00059 | 22.1 | 0.091 | **-0.00056** | **0.00016** | **53.9** | **0.001** | **-0.00323** | **0.00063** | **21.4** | **<0.001** |
| Visuospatial/executive | 0.00594 | 0.00554 | 10.4 | 0.308 | 0.00045 | 0.00184 | 30.9 | 0.808 | -0.00727 | 0.01040 | 14.7 | 0.495 |
| Naming | -0.00002 | 0.00196 | 41.3 | 0.910 | -0.00089 | 0.00063 | 27.7 | 0.167 | -0.00382 | 0.00254 | 16.9 | 0.152 |
| Attention | -0.00313 | 0.00272 | 24.0 | 0.261 | -0.00114 | 0.00087 | 34.2 | 0.199 | -0.00843 | 0.00510 | 23.5 | 0.112 |
| Language | -0.00514 | 0.00341 | 41.7 | 0.139 | -0.00191 | 0.00104 | 42.3 | 0.074 | -0.00264 | 0.00212 | 30.8 | 0.222 |
| Abstraction | 0.00057 | 0.00377 | 35.3 | 0.882 | -0.00096 | 0.00118 | 4.1 | 0.419 | -0.00607 | 0.00529 | 21.4 | 0.264 |
| Delayed memory | -0.01162 | 0.00689 | 38.5 | 0.100 | **-0.00473** | **0.00220** | **50.6** | **0.036** | **-0.01781** | **0.00835** | **30.4** | **0.041** |
| Orientation | -0.00290 | 0.00200 | 36.8 | 0.157 | **-0.00146** | **0.00063** | **50.9** | **0.025** | **-0.01032** | **0.00375** | **17.3** | **0.014** |
| CM-MMSE (n=51) |  |  |  |  |  |  |  |  |  |  |  |  |
| Total score | -0.00058 | 0.00039 | 59.8 | 0.138 | **-0.00027** | **0.00013** | **65.1** | **0.034** | **-0.00213** | **0.00053** | **17.1** | **<0.001** |
| Executive | **-0.00469** | **0.00128** | **55.2** | **<0.001** | **-0.00128** | **0.00432** | **60.3** | **0.004** | -0.00063 | 0.00189 | 13.2 | 0.743 |
| Memory | -0.00100 | 0.00091 | 44.9 | 0.278 | -0.00023 | 0.00030 | 58.0 | 0.435 | 0.00037 | 0.00166 | 46.0 | 0.823 |
| Attention and Calculation | 0.00060 | 0.00281 | 68.7 | 0.830 | -0.00031 | 0.00094 | 69.2 | 0.738 | -0.0079 | 0.00385 | 22.4 | 0.053 |
| Recall | 0.00001 | 0.00464 | 32.6 | 0.999 | -0.00095 | 0.00145 | 38.1 | 0.516 | -0.01381 | 0.00803 | 22.3 | 0.099 |
| Language and Praxis | -0.00006 | 0.00294 | 40.8 | 0.998 | -0.00048 | 0.00091 | 45.7 | 0.603 | **-0.01112** | **0.00532** | **22.6** | **0.048** |

*All models were adjusted for age, sex, education levels, disease onset age, disease duration, MDS-UPDRS-Ⅲ, Hoehn & Yahr stages, LEDD, HAMD and apathy (MDS-UPDRS-Ⅰ).

*Abbreviations:* CM-MMSE, China-Modified Mini-Mental State Examination; df, degree of freedom; FOG, Freezing of gait; HAMD, Hamilton Depression Scale; LEDD, levodopa equivalent daily dose; MDS-UPDRS, Movement Disorders Society Unified Parkinson’s Disease Rating Scale; MoCA, Montreal Cognitive Assessment; SE, standard error.
